# Transdermal Drug Delivery Enhancement by Compounds of Natural Origin

**DOI:** 10.3390/molecules161210507

**Published:** 2011-12-16

**Authors:** Lizelle T. Fox, Minja Gerber, Jeanetta Du Plessis, Josias H. Hamman

**Affiliations:** Unit for Drug Research and Development, North-West University, Private Bag X6001, Potchefstroom 2520, South Africa; Email: 12815268@nwu.ac.za (L.T.F.); Minja.Gerber@nwu.ac.za (M.G.); Jeanetta.duPlessis@nwu.ac.za (J.D.P.)

**Keywords:** natural, penetration enhancer, essential oil, terpene, polysaccharide

## Abstract

The transdermal route of administration offers an alternative pathway for systemic drug delivery with numerous advantages over conventional routes. Regrettably, the stratum corneum forms a formidable barrier that hinders the percutaneous penetration of most drugs, offering an important protection mechanism to the organism against entrance of possible dangerous exogenous molecules. Different types of penetration enhancers have shown the potential to reversibly overcome this barrier to provide effective delivery of drugs across the skin. Although certain chemical and physical skin penetration enhancers are already employed by the pharmaceutical industry in commercially available transdermal products, some skin penetration enhancers are associated with irritating and toxic effects. This emphasizes the need for the discovery of new, safe and effective skin penetration enhancers. Penetration enhancers from natural origin have become popular as they offer several benefits over their synthetic counterparts such as sustainable mass production from a renewable resource and lower cost depending on the type of extraction used. The aim of this article is to give a comprehensive summary of the results from scientific research conducted on skin penetration enhancers of natural origin. The discussions on these natural penetration enhancers have been organized into the following chemical classes: essential oils, terpenes, fatty acids and polysaccharides.

## 1. Introduction

The skin, as the largest organ of the body, serves as a protective layer of the underlying tissues such as muscles, ligaments and internal organs, shielding it from exogenous molecules as well as from mechanical and radiation-induced injuries. The skin also plays a role in immunology and metabolism, regulates body temperature, serves as an excretory organ through sebaceous and sweat glands and contains sensory nerve endings for the perception of touch, temperature, pain and pressure. The skin varies in color, thickness and presence of nails, hairs and glands between the different regions of the body, although all types of skin have the same basic structure [1,2].

The external surface of the skin is called the epidermis and consists of keratinized squamous epithelium. The next layer is the highly vascular dermis that nourishes and supports the epidermis and consists of a thick layer of dense, fibroelastic connective tissue which contains many sensory receptors. Underlying the dermis is the subcutaneous layer (or hypodermis) comprising of variable amounts of adipose tissue [2]. The skin has been investigated as a route to deliver drugs topically, regionally or systemically, but unfortunately dermal and transdermal drug delivery is often limited by poor drug permeability [3]. This low permeability can be mainly attributed to the most outer layer of the skin, called the stratum corneum, which serves as a rate-limiting lipophilic barrier against the uptake of chemical and biological toxins and the loss of water [4,5,6].

The stratum corneum is 15–20 μm thick [7] and is composed of 15–20 layers of flattened, densely packed, metabolically inactive cells, which are followed by several histologically distinguishable layers of closely packed cells. Furthermore, the epidermal cell membranes are so tightly joined that there is hardly any intercellular space through which polar non-electrolyte molecules and ions can diffuse [8]. The proteins and lipids of the stratum corneum form a complex interlocking structure, resembling bricks and lipid mortar [4]. The major lipids found in the stratum corneum include cholesterol and fatty acids [9]. Ceramides, in particular ceramide 2 and ceramide 5, play an important role in the stratum corneum’s overall lipid matrix organization and skin barrier function [10]. The ceramides are tightly packed in lipid layers due to the strong hydrogen bonding between opposing amide headgroups. This indicates a transverse organization in addition to the lateral orthorhombic chain organization of ceramide molecules. This hydrogen bonding is responsible for the strength, integrity and barrier properties of the lipid layers in the stratum corneum [11].

**Figure 1:**
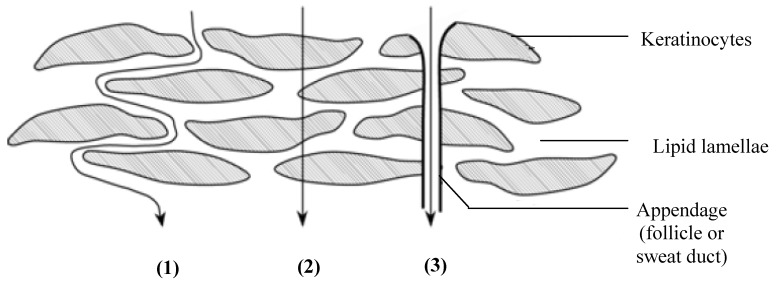
Drug permeation routes across the skin: (**1**) intercellular diffusion through lipid lamellae; (**2**) transcellular diffusion through the keratinocytes and lipid lamellae; and (**3**) diffusion through appendages such as the hair follicles and sweat glands (with permission from [12]).

The different routes by which a molecule can cross the stratum corneum include the transcellular, intercellular and appendageal (*i.e.*, through the eccrine/sweat glands or hair follicles) routes (Figure 1). The latter route is, however, considered to be insignificant partially due to the appendages occupying only a relatively low surface area [7].

Transdermal drug delivery offers the following advantages over oral administration: (1) peak and valley levels in the serum are avoided; (2) first-pass metabolism is avoided and the skin metabolism is relatively low; (3) less frequent dosing regimens is needed due to the maintenance and longer sustainability of zero-order drug delivery and (4) less inter-subject variability occurs [13]. Other advantages listed include aspects such as the accessibility of the skin; a relatively large surface area for absorption and the fact that it is non-invasive make it more patient compliant [14].

The drawback of the transdermal route of drug administration is the barrier presented by the stratum corneum that hampers drug permeation. This layer is very selective with respect to the type of molecule that it allows to be transported through the skin; therefore, only molecules with specific physico-chemical properties can cross the skin sufficiently [14]. This causes the range of potential drugs that can be administered transdermally to be very small, which highlights the need for incorporation of penetration enhancers into formulations that could assist in the effective delivery of a larger variety of drugs [5]. Both chemical and/or physical approaches can be used to enhance the penetration of drug molecules across the skin [15].

The properties of an ideal skin penetration enhancer include the following: (1) it should be odorless and colorless; (2) it should be specific in its mode of action; (3) it should be pharmacologically inert; (4) it should be compatible with drugs and other excipients; (5) it should be chemically and physically stable; (6) it should be non-allergenic, non-irritant and non-toxic; (7) its action should be reversible and (8) it should give a rapid effect for a predictable duration of time [16,17].

The site of action of the chemical skin penetration enhancers is located in the stratum corneum [18]. Chemical enhancers can be divided into two broad categories: Those that change partitioning into the stratum corneum and those that influence diffusion across the stratum corneum [19]. Examples of chemical penetration enhancers include sulfoxides (dimethylsulfoxide or DMSO), alcohols (ethanol), polyols (propylene glycol), alkanes, fatty acids (oleic acid), esters, amines and amides (urea, dimethylacetamide, dimethylformamide, pyrrolidones), terpenes, cyclodextrins, surfactants (non-ionic, cationic, anionic) and Azone^®^ [18,20].

It was proposed that skin penetration enhancers may act by one or more of three potential mechanisms according to the lipid-protein-partitioning theory. Firstly, penetration enhancers can alter the intercellular lipid structure between the corneocytes to increase diffusivity. Secondly, they can modify intracellular protein domains within the horny layer. Thirdly, they may increase the partitioning of the drug into the skin tissue [21].

The aim of this review article is to summarize and critically analyze scientific literature on penetration enhancers of natural origin with reference to their proposed mechanisms of action, effectiveness to deliver drugs across the skin and their shortcomings. The categories of natural skin penetration enhancers that are discussed include essential oils, isolated terpenes (from essential oils), fixed oils (or fatty acids) and complex polysaccharides.

## 2. Essential Oils

Essential oils are volatile, odoriferous substances found in the flowers, fruit, leaves and roots of certain plants. The extraction of these odoriferous compounds from plants has been an important occupation for over two thousand years [22]. The differences between volatile oils (or essential oils) and fixed oils (or fatty acids) are listed in Table 1.

**Table 1:** Differences between essential and fixed oils [23].

Essential oils	Fixed oils/Fatty acids
Distilled from different plant parts	Pressed from seeds
Very important to the plant’s life processes, although not involved in seed germination and early growth	Not so important for the plant’s life processes, although it is needed for seeds to germinate and sprout
Relatively small molecules built from rings and short chains	Relatively large molecules with large molecular sizes built from long carbohydrate chains
Volatile and aromatic	Non-volatile and non-aromatic
Circulate all through the plants and can diffuse through tissues, cell walls and membranes	Do not circulate in plants, diffuse through tissues or cell walls and membranes
Does not rot or go stale	Can rot and go stale
Can be anti-septic, anti-parasitic, anti-viral, anti-fungal and anti-bacterial	No anti-septic, anti-parasitic, anti-viral, |anti-fungal and anti-bacterial functions

Essential oils are complex mixtures of many diverse and unique chemical compounds [24]. According to numerous reports [22,25,26] essential oils consist of compounds which can generally be classified as:

– nitrogen- and sulphur-containing compounds (e.g., allyl isothiocyanate found in mustard oil);
– aromatic compounds, which are benzene derivatives (e.g., eugenol which is the main constituent of clove oil);
– terpenes (e.g., 1,8-cineole in eucalyptus oil) and terpenoids; and
– miscellaneous compounds (includes long-chain unbranched substances).

The skin penetration enhancing effect of several individual terpenes (discussed in Section 3 below) isolated from essential oils has been thoroughly investigated, while less data pertaining to their combinations either artificial or in natural form as essential oils exist [27].

### 2.1. Niaouli Oil

Niaouli oil is extracted through steam distillation from the leaves and twigs of *Melaleuca quinquenervia*, which is part of the Myrtaceae (Myrtle) family [23,24]. This oil is used for treating respiratory/sinus and urinary tract infections, allergies and hypertension [24]. Its key constituents is 55–70% 1,8-cineole (oxide) and limonene (monoterpene), 7–15% α-pinene (monoterpene), 2–6% β-pinene (monoterpene) and 2–6% viridiflorol (sesquiterpene) [23,24].

*In vitro* studies were performed using hairless mouse skin to determine the penetration enhancement effect of different essential oils at a 10% (w/w) concentration in propylene glycol on estradiol as model drug. Niaouli essential oil proved to be more effective than cajuput-, cardamom-, melissa-, myrtle- and orange essential oils for enhancing the transdermal penetration of estradiol. Thereafter, the four main terpene components of niaouli oil were investigated namely 1,8-cineole, α-pinene, α-terpineol and *d*-limonene individually at a 10% (w/w) concentration in propylene glycol and as two mixtures with the following compositions: Mixture 1 contained 62% 1,8-cineole, 20% α-pinene, 10% α-terpineol, 7.4% *d*-limonene and mixture 2 contained 69.2% 1,8-cineole, 22.5% α-pinene, and 8.3% *d*-limonene. Between the individual terpenes, 1,8-cineole was the best skin permeation promoter for estradiol. Both mixtures of the terpenes showed a similar lag time as that obtained by niaouli essential oil and a relatively high permeation enhancement effect, although significantly lower than that obtained for the whole niaouli essential oil. These results therefore showed that undefined phytoconstituents present at low concentrations in the whole niaouli essential oil may considerably increase its penetration enhancing activity [27].

It was further demonstrated that the same essential oils (such as niaouli oil) from different plant sources do not necessarily yield similar skin penetration enhancement results. Niaouli oils [10% (w/w)] from four different sources increased the transdermal flux of estradiol through hairless mouse skin from 41.50- to 84.63-fold compared to the control group, which consisted of vehicle containing propylene glycol and estradiol. The permeation values of estradiol for three of the niaouli oils tested did not differ significantly, although these three proved to be significantly superior in terms of transdermal penetration enhancement to the other niaouli oil tested [28]. These results emphasize the fact that biological effects of plant materials can vary from one source to another due to reasons such as differences in chemical composition of plants. It is therefore essential to chemically characterize plant materials that are tested for biological effects.

### 2.2. Eucalyptus Oil

Eucalyptus oil can be obtained from numerous species of the Myrtaceae family, which includes *Eucalyptus citriodora*, *Eucalyptus dives*, *Eucalyptus globules*, *Eucalyptus polybractea* and *Eucalyptus radiata*. The oil is extracted by steam distillation from the leaves. Generally, the key phytochemical constituents between the different species vary as can be seen in Table 2 [24].

**Table 2:** Key constituents of Eucalyptus essential oils from different natural sources [23,24].

Source of eucalyptus essential oil	Key constituents
*Eucalyptus citriodora*	Citronellal (75–85%)
	Neo-isopulegol and isopulegol (0–10%)
*Eucalyptus dives*	α- and β-Phellandrene (23–30%)
	Piperitone (35–45%)
	*p-*Cymene (6–10%)
	α-Thujene (2–6%)
	Terpinene-4-ol (3–6%)
*Eucalyptus globules*	1,8-Cineole (58–80%)
	α-Pinene (10–22%)
	Limonene (1–8%)
	*p*-Cymene (1–5%)
	*trans*-Pinocarveol (1–5%)
	Aromadendrene (1–5%)
	Globulol (0.5–1.5%)
*Eucalyptus polybractea*	1,8-Cineole (60–80%)
	*l*-Limonene (1–5%)
	*p*-Cymene (1–2%)
	α-Pinene (1–2%)
*Eucalyptus radiata*	Eucalyptol (60–75%)
	α-Terpineol (5–10%)
	*l*-Limonene (4–8%)
	α-Pinene (8–12%)

The model drug, 5-fluorouracil, was used to investigate the penetration enhancing activities of eucalyptus, chenopodium, ylang ylang and anise essential oils through excised human skin. Eucalyptus and chenopodium oils were the most effective drug permeation enhancers that exhibited almost a 30-fold increase in drug permeability coefficient followed by ylang ylang with an approximately 8-fold increase and anise oil proved to be the least effective permeation enhancer of the essential oils investigated in this study [29]. Further studies conducted showed that ylang ylang and anise essential oils had mild accelerating effects on drug flux across skin [30]. Furthermore, eucalyptus and chenopodium whole oils proved to be less effective as skin penetration enhancers than certain isolated terpenes from these oils. This could be attributed to the fact that the active constituents in the oils are not at maximum thermodynamic activity [30]. In addition, other phytoconstituents in the whole essential oil may reduce the skin penetration effect of these terpenes by means of different physical and chemical mechanisms.

Another penetration study on full-thickness human skin showed that eucalyptus oil enhanced the penetration of chlorhexidine (2% (w/v)) into the dermis and lower layers of the epidermis. When chlorhexidine was combined with 70% (v/v) isopropyl alcohol and 10% (v/v) eucalyptus oil, the skin penetration of the drug was significantly enhanced 2 min after application compared to a solution of chlorhexidine/isopropyl alcohol alone [31].

### 2.3. Alpinia oxyphylla Oil

*Alpinia oxyphylla* is a member of the ginger (Zingiberaceae) family and is used in oriental herbal medicine [32]. Essential oils from *A. oxyphylla *were extracted and subsequently divided into a lower-polarity fraction and a higher-polarity fraction [3]. The lower-polarity fraction contained eight sesquiterpenes (including estragol, copaene, 1*H*-cycloprop[e]azulene, himachal-2,8-diene, azulene, octahydro-1,8-dimethyl-7-(2-methylethenyl)-naphthalene, β-bisabolene, α-panasinsen), which were mostly hydrocarbon constituents except for the oxygenated constituent estragol and one cyclic monoterpene (*p*-cymene). The high-polarity fraction consisted of seven sesquiterpenes (including 1*H*-cycloprop[e]azulene, octahydro-1,8-dimethyl-7-(2-methylethenyl)-naphthalene, α-panasinsen, germacrene B, humulene 6,7-epoxide, *cis*-α-copaene-8-ol and nootkatone) with the latter three oxygenated sesquiterpenes making up most of the fraction [3].

It was found during an *in vitro* study with Franz diffusion cells using dorsal skin of Wistar rats that the high-polarity fraction of *A. oxyphylla* essential oil was more efficient in enhancing the skin permeation of indomethacin at concentrations of 3 and 5% than the lower-polarity fraction. There was, however, no significant difference in the flux of indomethacin between the different concentrations [3].

After pre-treatment with the two fractions of the essential oil [3% (v/v)] in carboxymethyl cellulose hydrogels (for 1 or 2 h) the permeation of indomethacin was significantly enhanced. The lag-time in drug penetration was also found to be reduced with pre-treatment. After pre-treatment with the essential oils, the skin deposition of indomethacin was also enhanced nearly 5-fold indicating that direct action on the skin governs the enhanced absorption of indomethacin rather than the release behavior of the vehicle. The essential oils showed a higher affinity for the lipophilic stratum corneum and may have reduced the polarity of the stratum corneum thereby enhancing the permeation of the lipophilic indomethacin into the skin [3].

During *in vivo* studies both fractions of *A. oxyphylla* essential oil significantly increased the skin uptake of indomethacin, although the high-polarity fraction showed greater enhancement which may demonstrate that the oxygenated sesquiterpenes exhibit greater ability to enhance skin uptake than the hydrocarbon sesquiterpenes. The *in vivo* studies showed that transepidermal water loss changes were negligible, indicating limited disruption of the intercellular routes by these drug permeation enhancers. The *A. oxyphylla* essential oils showed no irritancy and/or toxicity with the high-polarity fraction showing lower irritation than the low-polarity fraction. It was concluded that the main mechanism of the skin penetration enhancement effect by *A. oxyphylla* essential oils is due to the increase in the skin-vehicle partitioning. It was also suggested that the enhancing effect of the *A. oxyphylla* essential oils can be attributed to the combined effect of the different chemicals [3].

### 2.4. Turpentine Oil

Turpentine oil is obtained after distillation of the resin that is secreted by conifers (*Coniferae* spp). The use of turpentine oil dates back to the Ancient Greeks and is one of the most common essential oils [22]. Turpentine oil showed an additive effect on the skin permeation rate of flurbiprofen when it was added to an optimized co-solvent mixture of propylene glycol-isopropyl alcohol (30:70% (v/v)). A maximum transdermal penetration rate was obtained with turpentine oil at a concentration of 5% (v/v) and was found to be significantly more effective than tulsi oil at the same concentration. This is probably due to an increased disruption of the stratum corneum which is normally caused by terpenes, although it caused minor skin irritation. When compared to the binary solvent vehicle alone, 5% (v/v) turpentine oil had a significantly lower lag time for flurbiprofen flux across the skin [17].

### 2.5. Sweet Basil and Tulsi Oil

Sweet basil oil is obtained with steam distillation of the leaves, stems and flowers of *Ocimum basilicum* from the Lamiaceae or Labiatae (mint) family. This essential oil has numerous medicinal properties such as anti-inflammatory, muscle relaxant, anti-spasmodic, anti-viral and anti-bacterial. The key constituents include: methylchavicol (estragol) (40–80%, phenol), linalol (5–10%, alcohol), 1,8-cineole (1–7%, oxide) and eugenol (1–10%, phenol) [23,24].

The influence of *O. basilicum* or basil essential oil extract on the permeation of drugs through the skin was investigated *in vitro* by employing Franz diffusion cells using the dorsal skin of Wistar rats. Both *in vitro *and *in vivo* studies were used to determine the amount of drug uptake within the skin reservoir. Low-polarity and high-polarity fractions of basil essential oil were obtained. The low-polarity fraction contained predominantly estragol (aromatic ether), followed by squalene (triterpene) and the sesquiterpenes α-bergamotene and θ-muurolene. Most of the components were hydrocarbon constituents, with estragol being the only oxygenated constituent. Phytol (diterpene) was the highest/most occurring compound in the high-polarity fraction. Other compounds also present in the high-polarity fraction included the oxygenated terpenes *d*-linalool, estragol and butylated hydroxytoluene. A hydrocarbon sesquiterpene (+)-*epi*-bicyclosesquiphellandrene were also present. The authors found that both fractions enhanced the skin permeation of indomethacin, with the low-polarity fraction proving to be more efficient which indicated that essential oils with a lower polarity enhance more effectively [33].

*In vitro* permeation studies after pre-treatment with the sweet basil oil significantly enhanced the permeation of indomethacin and its uptake into the skin reservoir. It was suggested that basil essential oil work by increasing drug partitioning into the stratum corneum and by disrupting the skin morphology. *In vivo* microdialysis studies indicated that the amount of indomethacin in the subcutaneous region was generally higher for the low-polarity fraction compared to the control group; whereas the indomethacin concentration in the microdialysis dialysate were negligible for the high-polarity fraction group. The enhancing activity of basil essential oils on hydrophilic 5-fluorouracil was found to be lower compared to that for the more lipophilic model compound, indomethacin. *In vivo* skin deposition experiments in contrast to the *in vitro* experiments showed that the high-polarity fraction had a greater ability to retain the drug within the skin than the low-polarity fraction [33].

It was found that basil oil was the most effective penetration enhancer for the hydrophilic drug labetolol hydrochloride across rat abdominal skin, followed by camphor, geraniol, thymol and clove oil. A synergistic effect of the vehicle system (ethanol-water, 60:40) and terpenes were observed with the overall flux values. When terpenes are present, a competitive hydrogen bonding between ceramides and terpenes arise, causing the tight junctions of lipid layers to loosen and create new pathways for molecular permeation. Terpenes with a more electronegative alcoholic group (such as in basil oil) interact with the amide groups of the ceramides more competitively than the terpens with a less electronegative carbonyl group, such as camphor that contains a ketone oxygen atom. In contrast to these findings, it was determined that geraniol, thymol and clove oil showed a lower enhancement ratio than camphor, despite these oils containing more electrophilic alcoholic oxygen atoms. This indicates that the physico-chemical properties of the permeant molecules in addition to that of the enhancer molecules, significantly alters the permeation of a molecule across the skin. Lag time of labetolol hydrochloride was also significantly decreased in the following order: camphor < basil oil < geraniol < thymol < clove oil < vehicle < water [34].

As mentioned before, the lipid layers in the stratum corneum are held together by both a lateral and transverse hydrogen bonding network. In order to push molecules across the transverse hydrogen bonding, higher activation energy is needed. When the stratum corneum is treated with terpenes, it is thought that the barrier is disrupted due to competitive hydrogen bonding between the lipids and terpenes which leads to lower activation energies of molecules to diffuse across the skin. It was concluded that basil oil created new polar pathways in the stratum corneum lipids by which labetolol hydrochloride could permeate [34].

Experiments showed non-toxicity of the basil essential oil as it did not increase the prostaglandin E_2_ (PGE_2_) level of cultured keratinocytes and fibroblasts. Basil essential oil was also found not to increase transepidermal water loss, thereby illustrating low irritancy [3].

Tulsi oil (obtained from *Ocimum sanctum*) and turpentine oil was investigated for their penetration enhancing effects on the model drug flurbiprofen. When added to an optimized binary solvent mixture of propylene glycol:isopropyl alcohol [30:70% (v/v)], tulsi oil further enhanced the transdermal permeation rate of flurbiprofen. Tulsi oil at a concentration of 5% (v/v) showed the highest flux enhancement factor and the lag time was significantly lower. The enhancement of the transdermal permeation rate of flurbiprofen with tulsi oil was thought to be accomplished by modifying the barrier properties of the stratum corneum [17].

When skin is treated with 5% (v/v) tulsi oil in propylene glycol-isopropyl alcohol [30:70% (v/v)] the stratum corneum shows widespread disruption with condensation of the normally stratified corneal layers. The epidermal thickness increases from a normal 2–3 cell layers to 4–5 cell layers; whereas the dermis does not show any significant changes [17].

### 2.6. Cardamom Oil

Cardamom oil is distilled from *Elettaria cardamomum* (cardamom) which is part of the ginger (Zingiberaceae) family. It is used as an anti-spasmodic, expectorant and anti-parasitic agent. The key constituents of this oil includes: α-terpinyl acetate (45–55%, ester), 1,8-cineole/eucalyptol (16–24%, oxide), linalol (4–7%, alcohol), linalyl acetate (3–7%, ester) and limonene (1–3%, monoterpene) [23,24].

Previous studies indicated that an acetone extract of cardamon seed (*E. cardamomum*) enhanced the dermal penetration of prednisolone across abdominal mouse skin. Two fractions obtained from this acetone extract were further separated and the compounds were identified as acetyl terpineol (*d*-α-terpinyl acetate) and terpineol (*d*-α-terpineol). The two fractions as well as the further separated compounds all proved to be better skin penetration enhancers of prednisolone than Azone^®^ (1-dodecylazacycloheptan-2-one) [35].

In another study where cardamom oil was distilled from the seed of *Amomum cardamomum*, it was found with an *in vitro* permeation study through rabbit abdominal skin that the oil enhanced the penetration of indomethacin, diclofenac and piroxicam [36]. It was determined that the enhancing effect of cardamom depended on its concentration, with a 1% (v/v) concentration being more effective than 0.5% (v/v). The penetration index of the drugs was determined as the ratio of the flux of formulation containing enhancer, divided by the flux of the control formulation without enhancer. The penetration index at both pH 5.8 and pH 7.4 with 1% cardamom oil was the highest for piroxicam, followed by indomethacin and then diclofenac. At a concentration of 1% (v/v) cardamom oil, a shorter lag time was observed for the permeation of indomethacin and diclofenac across the skin. Their results indicated that cardamom oil has an enhancing effect which is dependable on its concentration, the pH of the solvent and the physicochemical properties of drug rather than the solubility of the drug in the solvent system [36].

Further *in vivo* studies showed that a 30 min pre-treatment of rabbit abdominal skin with 5% cardamom oil in an alcohol-water vehicle (1:1) increased the peak area of the plasma concentration time curve of piroxicam (AUC 0–24 h) 67.09-fold when compared to non-treatment. In addition, an absolute bioavailability of 83.23% was obtained. Results after a 60 min pre-treatment were not significantly different from that after a 30 min pre-treatment [37].

### 2.7. Peppermint Oil

Peppermint oil is extracted by steam distillation from the stems, leaves and flower buds of the plant *Mentha piperita* from the Lamiaceae or Labiatae family. The key constituents of the oil include: menthol (34–44%, phenolic alcohols), menthone (12–20%, ketone), menthofurane (4–9%, furanoids), 1,8-cineole (eucalyptol, 2–5%, oxide), pulegone (2–5%, ketone) and menthyl acetate (4–10%, ester) [23,24]. The oil is used to relieve pain, to control appetite, to stimulate digestion/gallbladder function and as anti-inflammatory, anti-tumoral, anti-viral, anti-bacterial and anti-parasitic agent [24].

Three natural oils, namely peppermint, eucalyptus and tea tree oil were investigated [38] to determine how they affect human skin integrity. *In vitro* permeation studies on human breast or abdominal skin was performed by applying the natural oils in 0.1%, 1.0% or 5.0% (v/v) concentrations in aqueous solutions containing 1% (v/v) Tween, 0.9% (w/v) NaCl and triturated water (^3^H_2_O) [38]. The flux of ^3^H_2_O is indicative of the integrity of the skin with a high flux value suggesting damage to the skin [38]. This study indicated that with an increase in the concentrations of the three oils, the flux of ^3^H_2_O increased, while at the lowest concentration a decrease in the percutaneous penetration of ^3^H_2_O was observed signifying a protective effect. Peppermint oil showed the most significant effect on skin integrity and was therefore studied further to determine its effect on the percutaneous penetration of benzoic acid at different concentrations. Results showed that the peppermint oil was generally protective against the penetration of the hydrophilic drug at the lower concentrations of 0.1% and 1.0% (v/v) [38].

### 2.8. Fennel Oil

Fennel oil is obtained from steam distillation of the crushed seeds of *Foeniculum vulgare*, which is part of the Apiaceae or Umbelliferae family. Its main components is 60–80% *trans-*anethole (phenolic ester), 12–16% fenchone (ketone), linalol (alcohol), 3–5% α-pinene (monoterpene) and 2–5% methyl chavicol (phenol) [23,24]. It can be used as a digestive aid, anti-septic, anti-spasmodic, analgesic and anti-parasitic agent. It also has anti-inflammatory, anti-tumoral characteristics and can increase metabolism [24].

After pre-treatment with a series of essential oils at a concentration of 10% (w/w) in propylene glycol, fennel oil was found to be the most effective enhancer for the percutaneous penetration of trazodone hydrochloride, which was followed by eucalyptus oil, citronella oil and mentha oil. Propylene glycol pre-treatment itself also significantly enhanced the permeation of trazodone hydrochloride; nevertheless pre-treatment with 10% fennel oil in propylene glycol showed an enhancement ratio of 9.25 compared to the control. The phytochemicals with variable physico-chemical properties and molecular weights present in the different essential oils may be the cause of differences in the permeation enhancement ratios between the oils. *Trans*-anethole and 1,8-cineole have low boiling points and molecular weights which may contribute to the higher enhancement ratio of fennel oil and eucalyptus oil [39].

### 2.9. Black Cumin Oil

Black cumin essential oil is obtained with steam distillation from the seeds of *Cuminum cyminum* of the Apiaceae or Umbelliferae family. It can be used as an immune stimulant, digestive aid, liver protectant, antioxidant, anti-inflammatory, anti-tumoral and anti-viral. Its major components include: Cuminaldehyde (16–22%, aldehyde), γ-terpinene (16–22%, monoterpene), β-pinene (12–18%, monoterpene), *p*-mentadienal (25–35%, aldehydes) and *p*-cymene (3–8%, monoterpenes) [23,24]. Black cumin oil was found to be a better penetration enhancer with an enhancement factor of 6.40 for the model lipophilic drug, carvedilol, when compared to clove oil, eucalyptus oil, tulsi oil, oleic acid and Tween 80. Thermal analysis (Differential Scanning Calorimetry or DSC) of 5% (v/v) black cumin oil in isopropyl alcohol indicated that the oil has the capability to extract lipids (fluidizing the skin) and cause α-keratin denaturation that alters the skin protein composition. This creates a passage for the drug to cross the dermis. Fourier Transform Infrared Spectroscopy (FTIR) confirmed that black cumin oil alters the permeability of the skin by extracting lipids and by hydrogen bonding which affect other hydrogen bonds between the ceramides [40].

## 3. Terpenes

‘Terpene’ is a term used to describe a compound that is a constituent of an essential oil that does not have an aromatic character and contains carbon and hydrogen atoms with/without oxygen. In some cases this term is also given to compounds which are closely related to the natural terpenes, although they are not of natural origin [22,41].

Terpenes are well recognized penetration enhancers for drug permeation across the human skin and have been receiving considerable interest in the pharmaceutical industry for this application [42,43]. They are in general clinically acceptable and relatively safe skin penetration enhancers for both lipophilic and hydrophilic drugs [43]. Terpenes are one of the most extensively studied classes of chemical penetration enhancers. The various classes, different physico-chemical properties and different potential mechanisms of action make the terpenes a promising group of compounds for transdermal delivery of drugs with a wide range of physico-chemical properties [41].

**Figure 2:**
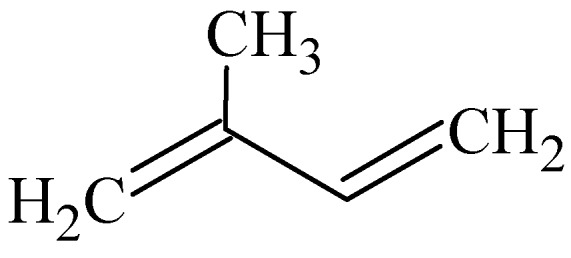
Isoprene unit.

The carbon skeletons of most terpenes can be built up by the union of two or more isoprene (C_5_H_8_) residues (Figure 2) united in a head-to-tail manner. Terpenes can be classified according to the presence of the number of isoprene units in the molecule as illustrated in Table 3 [22,44].

**Table 3:** Classification of terpenes according to the number of isoprene units [41,44].

	Number of isoprene units	Number of carbon atoms
Monoterpenes	2	C_10_
Sesquiterpenes	3	C_15_
Diterpenes	4	C_20_
Sesterterpenes	5	C_25_
Triterpenes	6	C_30_
Tetraterpenes	8	C_40_
Polyterpenes	>8	>C_40_

All terpenes can be subdivided into ‘acyclic’, ‘monocyclic’ and ‘bicyclic’ categories based on the number of carbon-rings the structure of the terpene contains [22,41]. Acyclic monoterpenes can be regarded as derivatives of 2,6-dimethyloctane, while monocyclic monoterpenes are derivatives of cyclohexane containing isopropyl substituents. Bicyclic monoterpenes are arranged in more than one aromatic ring, which contains the same number carbon atoms. Sesquiterpenes are present as acyclic, monocyclic, bicyclic, tricyclic and tetracyclic structures and are mostly found in higher plants [22,41].

Terpenes of natural origin have a ‘Generally Regarded As Safe’ (GRAS) status with the Federal Drug Administration of the United States of America, which offers advantages over other traditional enhancers such as alcohols, fatty acids, Azone^®^, sulfoxides and pyrrolidones [41]. In general, they have low systemic toxicity and skin irritancy in addition to having good penetration enhancing abilities [5]. A classification of the different terpenes described in the sections below for their skin penetration enhancement properties can be found in Table 4.

**Table 4:** Classification of terpenes that have been investigated for their skin penetration enhancement effects.

**Class**	Example(s) of terpene	Source
**ACYCLIC MONOTERPENES (Alcohols)**	Geraniol and nerol	Geraniol is an unsaturated primary alcohol found in geranium and other essential oils. It is found as esters and as a glucoside, but mainly occurs in the free form. Nerol is the isomeric alcohol and is found in various essential oils, primarily in neroli and bergamot oils [22,41]. Palmarosa oil contains more geraniol than any other oil and for nerol it is catnip and rose oil [23].
** **	Linalol	Linalol is found as (+)- and (−)-forms in the oil of Linaloe (a plant found in Central America), but can also be found free and as esters in numerous other essential oils [22]. Rosewood oil contains more linalol than any other oil [23].
**MONOCYCLIC MONOTERPENES (Hydrocarbons)**	Limonene	The optically active limonene is widespread in nature and is found in its (+)- and (−)-forms in various essential oils such as bergamot, caraway, lemon and orange oils [22,41]. The signature oils for *d-*limonene and *l-*limonene is grapefruit and fleabane, respectively [23].
**MONOCYCLIC MONOTERPENES (Alcohols)** Alcohols related to α-terpineol	α-Terpineol	Found in many essential oils such as camphor, neroli and petitgrain oil [22]. The signature oil is lemon eucalyptus [23].
** **	β-Terpineol	Isomeric with α-terpineol, but is not isolated from natural sources. Found in commercial terpineol [22].
** **	γ-Terpineol	Second isomer of α-terpineol and is found in at least one essential oil and commercial terpineol [22].
**MONOCYCLIC MONOTERPENES (Alcohols) **Alcohols derived from thymol	Menthol	Menthol is a constituent of numerous peppermint oils and is found as its (−)-form [22,23].
**MONOCYCLIC MONOTERPENES (Alcohols)** Alcohols derived from carvacrol	Carveol	Carveol is found in caraway oil [22,23].
**MONOCYCLIC MONOTERPENES (Ketones)** Ketones related to menthone	Menthone	(−)-form is found in numerous peppermint oils, (+)-form also occurs naturally [22,41].
** **	Pulegone	Found in pennyroyal and many other essential oils as its (+)-form [22].
** **	*iso-*Pulegone	Often an accompaniment of pulegone in essential oils [22].
** **	Piperitone	Occurs in numerous eucalyptus oils as (+)- and (−)-forms [22].
**MONOCYCLIC MONOTERPENES (Ketones)** Ketones related to carvomenthone	Carvomenthone	Isomeric with menthone and is a saturated ketone. (−)-Form is found in numerous essential oils [22].
** **	Carvone Unsaturated ketone	Occurs in its (+)-, (−)- and (±)-forms and is the main constituent of caraway and dill oils [22]. It can also be found in spearmint oil [41].
**MONOCYCLIC MONOTERPENES (Oxides)**	1,8-Cineole	Widespread in essential oils, particularly in eucalyptus and wormseed oil [22,41].
**BICYCLIC MONOTERPENES (Hydrocarbons)**	α-Thujene	Found in numerous essential oils [22].
**BICYCLIC MONOTERPENES (Hydrocarbons)**	Car-3-ene	Found in several turpentine oils [22].
**BICYCLIC MONOTERPENES (Hydrocarbons)**	α-Pinene	Widespread in nature, found in most essential oils of *Coniferae*. It is the main constituent of turpentine oil. Secreted by conifers, turpentine oil consists of resinous material dissolved in turpentine oil [22].
** **	β-Pinene (Nopinene)	Isomeric with α-pinene [22]. Its signature oil is galbanum [23].
**BICYCLIC MONOTERPENES (Oxygenated derivatives)**	Verbenol, verbenone and verbanone	Verbenol and verbenone has been found in nature, with the latter being found in verbena oil [22]. The signature oil for verbenone is rosemary verbenone [23].
**BICYCLIC MONOTERPENES (Ketones – camphane group)**	Camphor	Not widely distributed in nature, is the major constituent of camphor oil, obtained from the leaves and wood of the camphor tree (*Cinnamomum camphora*) [22].
**BICYCLIC MONOTERPENES (Ketones – fenchane group**	Fenchone	Occurs as the optically active forms in fennel, thuja and cedar leaf oils [22,23].
**SESQUITERPENES (Alcohol)**	Farnesol	Widely distributed in flower oils, in particular those of the acacia, cyclamen and the rose [22].
** **	Nerolidol	Isomeric with farnesol and found in neroli oil [22].
** **	(−)-Guaiol	A crystalline alcohol found in guaiacum wood oil [22].
** **	(+)-Cedrol	Cedarwood oil [45].
** **	(−)-α-Bisabolol	Camomile oil [45].
**SESQUITERPENES (Hydrocarbon)**	Bisabolene	Widespread in nature, found in bergamot and myrrh oils. Also in many other essential oils [22].
** **	The Azulenes	All hydrocarbons are derived from azulene (C_10_H_8_), a parent hydrocarbon. Most of those attained from natural origin have the molecular formula C_15_H_18_. Azulenes is responsible for the blue color of certain essential oils, or when essential oils become blue/violet when undergoing processes which might result in dehydrogenation [22].
**(Unsaturated hydrocarbons)**	
** **	(+)-Longifolene	Tricyclic sesquiterpene found in the essential oil of *Pinus longifolia *[22].
** **	β-Caryophyllene	Main hydrocarbon constituent of clove oil [22].
** **	(+)-Aromadendrene	Eucalyptus oil [45].
** **	(+)-*β-*Cedrene	Cedarwood oil [45].
**ACYCLIC DITERPENES (Alcohol)**	Phytol	Found in rosemary oil [22,23].
**ACYCLIC TRITERPENES (Hydrocarbon)**	Squalene	It is found in the unsaponifiable fraction of shark liver oil and in several plant sources such as vegetable oils and several fungi [22]. Jasmine is the signature oil [23].

### 3.1. Limonene

**Figure 3:**
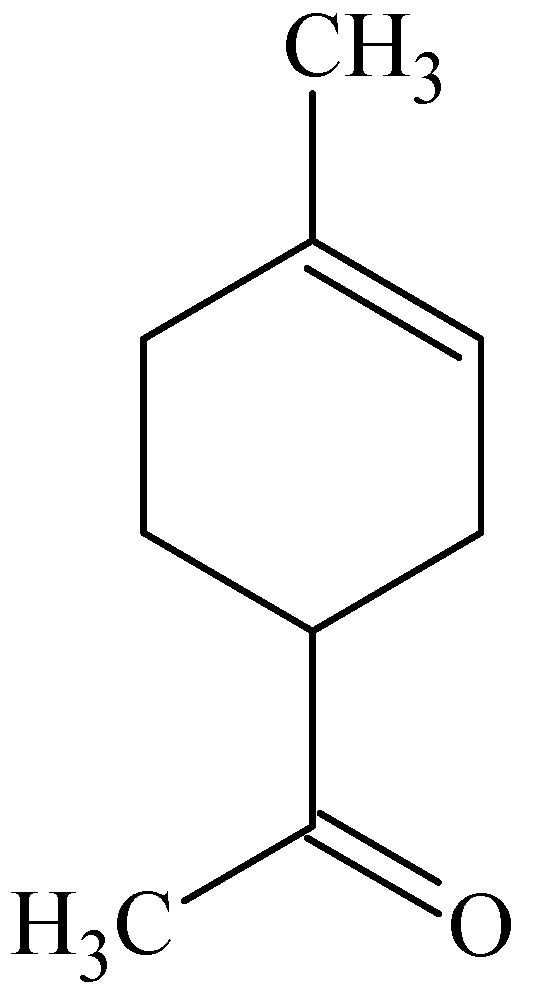
Chemical structure of (+)-limonene.

Limonene (Figure 3) was more effective than oxygenated linalool and cineole (in combination with propylene glycol) for improving the permeability of haloperidol across female human abdominal skin. Linalool and cineole showed only moderate enhancement and extended lag time, whereas limonene improved the permeability of haloperidol 26.5-fold and reduced the lag time of haloperidol transport across female human abdominal skin [46]. Limonene was also found to have a higher penetration value for dihydrotestosterone into hairless rat skin compared to oleic acid [47].

R-(+)-limonene showed a high ability to enhance *in vitro* percutaneous transport of sumatriptan across porcine skin after pre-treatment compared to the control (buffer). The results indicated that the highest skin penetration enhancing effect on sumatriptan was found for limonene, while more lipophilic penetration enhancing compounds (e.g., Span^®^ 20, α-bisabolol, oleic acid) and more hydrophilic penetration enhancing compounds (e.g., ethanol, polyethylene glycol 600, 1,8-cineole) showed lower capacity to increase sumatriptan’s transdermal flux [48].

In an investigation where terpenes from four different chemical classes, namely hydrocarbons (*d*-limonene), alcohols (geraniol), epoxides (α-pinene oxide) and cyclic ethers (1,8-cineole) were used to pre-treat third-degree burn eschar from abdominal and lower external burns, the permeation flux of the anti-microbial drug, silver sulphadiazine, was increased. The highest enhancement ratio was observed for limonene (about 9 times the normal flux), followed by geraniol (5.5 times), eucalyptus oil (4.7 times) and α-pinene oxide (4.3 times). Limonene was found to decrease the lag time significantly (20%), whereas the other terpenes showed a negligible increase in permeation lag-times. It was determined that the increased permeation of silver sulphadiazine can be attributed to the increased partition of the drug into the eschar. For intact skin both increased diffusion coefficient as well as partitioning play a role when using terpenes [49].

A membrane-moderated transdermal therapeutic system of nicardipine hydrochloride utilizing a reservoir containing 2% (w/w) hydroxyl propyl cellulose gel with 4% (w/w) limonene as penetration enhancer improved not only the bioavailability of the drug by 2.62 times, but also provided a prolonged steady state concentration of nicardipine hydrochloride [50].

### 3.2. Menthol

**Figure 4:**
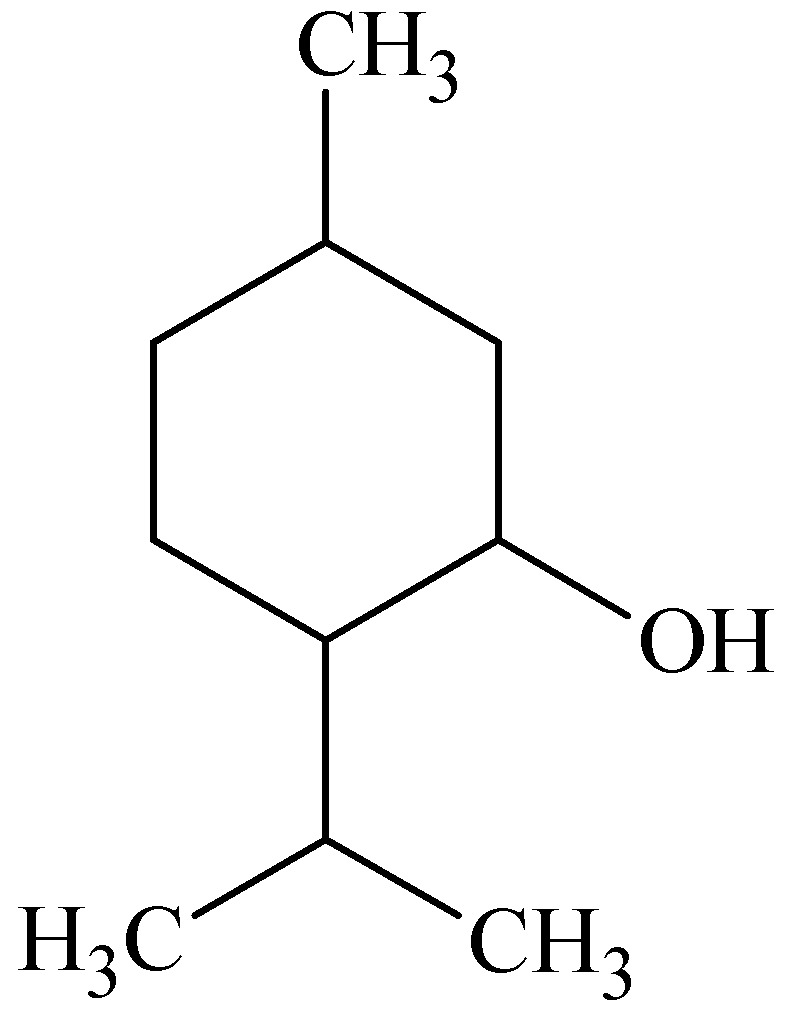
Chemical structure of menthol.

**Figure 5:**
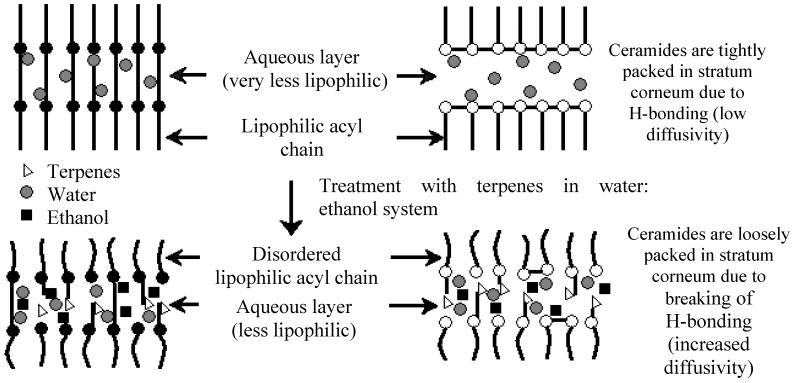
Mechanism by which terpenes act on the lipid bilayer of the stratum corneum (with permission from [51]).

Menthol (Figure 4) was found to be analogous to cineole in being the most effective penetration enhancer for imipramine hydrochloride in an ethanol-water (2:1) system through dorsal rat skin. This was followed by terpineol, menthone, pulegone and carvone [51]. A likely mechanism for the enhancement of imipramine hydrochloride permeation across the skin by terpenes (*i.e.*, menthol and terpineol) was proposed to be the disruption of the hydrogen bond network at the heads of the ceramides (Figure 5), which was supported by FT-IR data obtained. In the case of menthone, pulegone, carvone and cineole, however, only hydrogen bond accepting moieties (carbonyl or ether groups) are present, leading to less disruption of the hydrogen bond network between the ceramide heads. It was concluded that a key factor in the enhancement of the studied drug’s enhancement by terpenes is the hydrogen bond accepting and donating strength alongside self-association of terpene molecules [51].

Thymol, carvacrol, *trans-*anethole and linalool were found to enhance the transdermal transport of azidothymidine comparable or better than *l*-menthol. It was found during *in vitro* concentration optimization studies that a concentration of 5% of these penetration enhancers is most effective in enhancing the transport of azidothymidine through nude mouse skin [52]. The amount of azidothymidine retained in the skin was determined by *in vitro* transport studies with formulations containing 0–10% (w/w) enhancer and 30 mg/mL azidothymidine in isopropyl:water (60:40 (v/v)). These studies showed that there was no correlation between the amount of azidothymidine retained in the skin and the enhancer levels. This indicated that the enhancers increased the diffusion coefficient of the drug in the skin rather than affecting skin partitioning [52].

### 3.3. 1-8-Cineole

**Figure 6:**
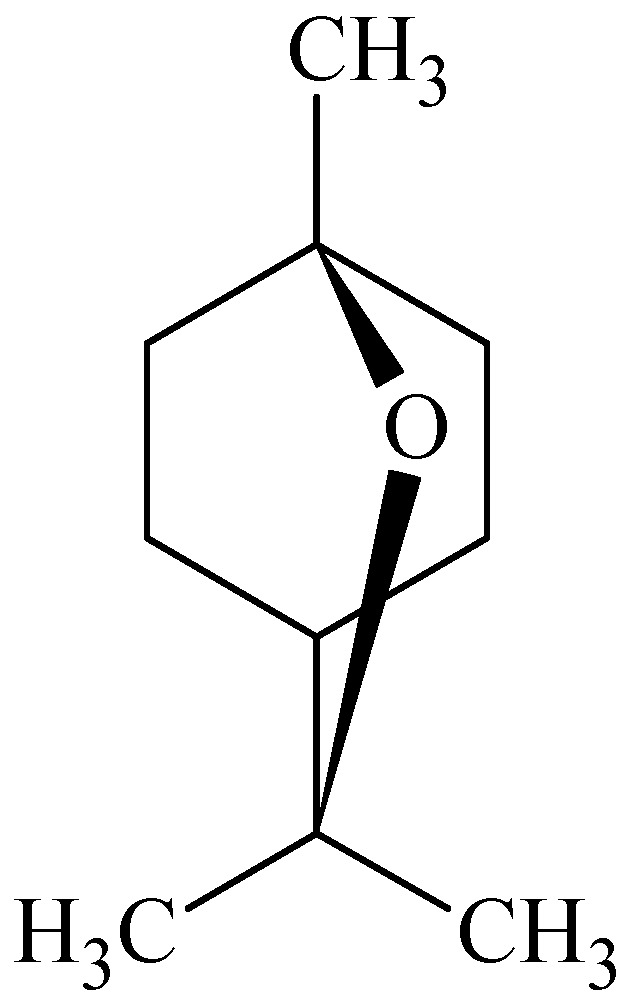
Chemical structure of 1,8-cineole.

Williams and Barry [30] assessed a series of cyclic terpenes from the broad chemical classes of hydrocarbons, alcohols, ketones and oxides for their possible skin penetration enhancing effects on 5-fluorouracil. It was found that the terpenes varied with their activities, but 1,8-cineole (Figure 6) caused an almost 95-fold increase in the enhancement of 5-fluorouracil making it the most effective skin penetration enhancing terpene. Hydrocarbons, which included *d*-limonene, α-pinene, 3-carene, showed the lowest activity with the latter of the three having the greatest enhancement ratio. Carveol proved to be the most effective terpene penetration enhancer from the alcohol group, which also included α-terpineol and terpinen-4-ol. Ketones studied included carvone, pulegone, piperitone and menthone with the latter being the most effective. It was shown that the terpene skin penetration enhancers disrupted the stratum corneum lipids thereby increasing diffusivity. No significant protein interaction or major partitioning alterations were observed [30].

The modes of action of three terpene penetration enhancers (*d*-limonene, nerolidol and 1-8-cineole) in human skin were also investigated by DSC and small-angle X-ray diffraction (SAXRD) [5]. The authors compared the effect of the terpenes with and without propylene glycol on the structure of the stratum corneum. The terpenes were tested undiluted and as saturated solutions in propylene glycol to ensure that thermodynamic activity was the same. Nerolidol was found to be completely miscible with propylene glycol and was applied as a 90% (w/w) solution. After a 12 h treatment, the uptake of *d*-limonene, 1-8-cineole and nerolidol into the stratum corneum was 8.90, 26.20 and 39.60% (w/w) dry tissue weight, respectively. Propylene glycol did not significantly alter *d*-limonene uptake, but significantly reduced uptake of 1-8-cineole and nerolidol into the stratum corneum. This is in contrast to previous arguments that propylene glycol may increase the uptake of the enhancer into the stratum corneum. It is postulated that the terpenes pool in the stratum corneum to form microdroplets in the intercellular lipid domain. Evidence obtained with DSC and SAXRD indicated that propylene-glycol/terpene synergy may produce enhanced lipid bilayer disruptions [5]. At physiological temperatures DSC studies provided proof that 1-8-cineole and nerolidol is lipid disruptive, which coincided with the results obtained by Williams and Barry [30]. On the other hand, no clear evidence was found to support disruption of the intercellular bilayers by *d*-limonene [5].

An *in vitro* study conducted on rat abdominal skin made use of film formulations of propranolol hydrochloride containing menthol, cineole and/or propylene glycol as penetration enhancers. Cineole proved to be superior to the other penetration enhancers at a concentration of 5% (w/w), followed by propylene glycol in combination with cineole. Increasing the concentration of cineole to 10% (w/w) when used as single penetration enhancer or in combination with propylene glycol showed the highest permeation rate of propranolol hydrochloride [53].

### 3.4. Carvone

**Figure 7:**
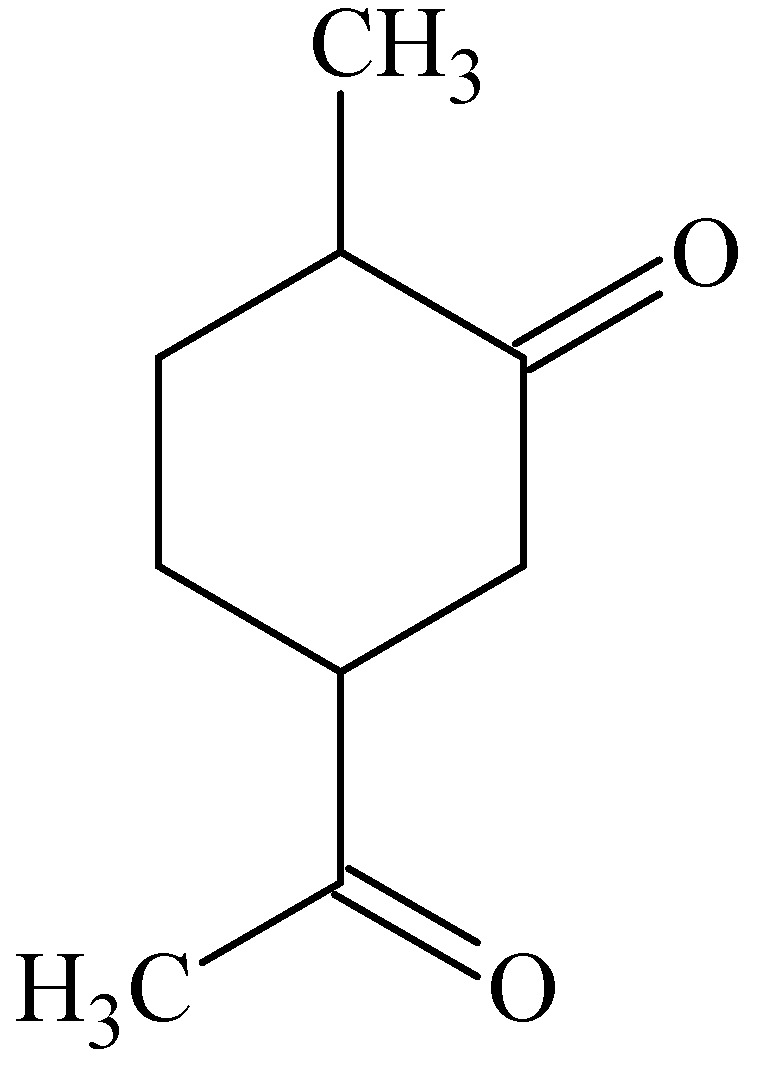
Chemical structure of (+)-carvone.

*In vitro* permeation studies across porcine epidermis were performed to investigate the enhancing effects of four cyclic terpenes namely carvone (Figure 7), 1-8-cineole, menthol and thymol. At a concentration of 5% (w/v) of these terpenes in combination with 50% (v/v) ethanol in water the transport of tamoxifen compared to the control of 50% (v/v) ethanol in water was increased. Carvone was the most effective terpene, followed by 1-8-cineole, thymol and menthol. It was found that thymol and menthol increased the partitioning of tamoxifen into the stratum corneum, while carvone and 1-8-cineole had no effect. Enhancement of the permeability coefficient by carvone and 1-8-cineole was therefore thought to be due to disruption of the stratum corneum lipids. Permeability enhancement of tamoxifen caused by menthol and thymol may thus be partially ascribed to improvement of partitioning of the drug into the stratum corneum [43].

Carvone at a concentration of 8% (w/w) was employed as a penetration enhancer in a membrane-moderated transdermal therapeutic system of nicardipine hydrochloride that was evaluated *in vivo*. The bioavailability of nicardipine hydrochloride was increased 3-fold with reference to an immediate-release capsule dosage form in healthy male volunteers [54].

### 3.5. Geraniol

**Figure 8:**
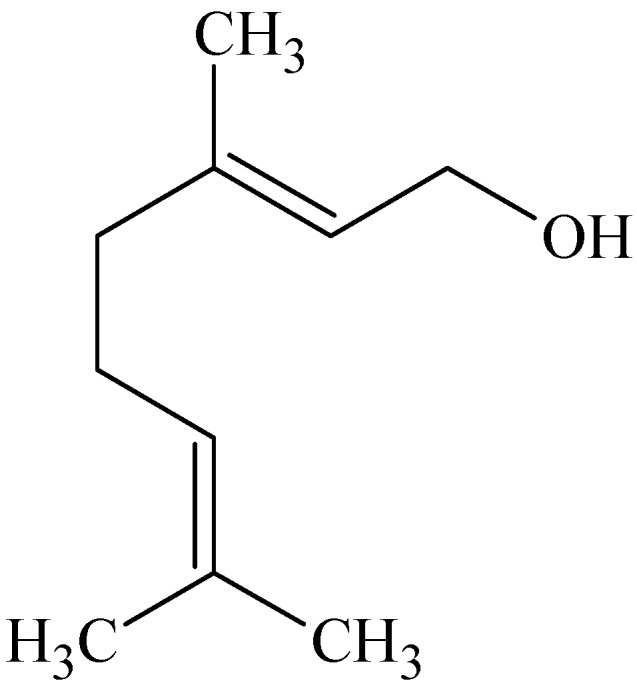
Chemical structure of geraniol.

Geraniol (Figure 8) is an acyclic monoterpene and has a *trans-*conformation with two double bonds [55]. The enhancing effect of this naturally occurring terpene as well as other terpenes which included *cis*-nerolidol, (−)-menthol, thymol, 1,8-cineole, menthone, (−)-fenchone and (+)-limonene were investigated [56]. *In vitro* percutaneous absorption of diclofenac sodium from carbomer gels containing propylene glycol across full-thickness abdominal male Wistar rat skin were examined. The results indicated that the alcohol terpenes were efficient accelerants for diclofenac sodium. Geraniol proved to be the best penetration enhancer with a nearly 20-fold increase in diclofenac sodium’s permeability coefficient. This was followed by nerolidol (14-fold increase) and menthol (11-fold increase). Thymol proved not to be as efficient as the aliphatic alcohols. A mild increase was seen with limonene (hydrocarbon terpene); whereas fenchone and menthone (ketone terpenes) were less effective and the oxide terpene (1,8-cineole) proved to be a poor accelerant. The acyclic terpenes, geraniol and nerolidol, demonstrated the best enhancing effects of the alcohols tested. The presence of definitive hydrocarbon tail groups besides a polar head group makes the structures of these terpenes suitable for disrupting the lipid packing of the stratum corneum [56].

Typically, the transdermal absorption of hydrophilic drugs is better improved by terpenes with polar functional groups, whereas the absorption of lipophilic drugs is more enhanced by hydrocarbon terpenes. However, it was found that the hydrocarbon terpene was more effective than the ketones and oxide terpene. The authors suggested that it can be ascribed to the lower thermodynamic activity of the ketones in the gels. The physicochemical nature of the drugs and terpenes as well as the vehicle in which they are formulated plays a major role in drug absorption through the skin [56].

Geraniol proved to be the most effective penetration enhancer of eleven monoterpenes including (+)-limonene, (−)-menthone, (+)-terpinen-4-ol, α-terpineol, 1,8-cineole, (+)-carvone, (−)-verbenone, (−)-fenchone, *p*-cymene, (+)-neomenthol and geraniol investigated to increase permeation of caffeine through hairless mouse skin. Other model drugs studied included hydrocortisone and triamcinolone acetonide to give a range of drugs with different lipophilicities. The terpenes were applied at 0.4 M in propylene glycol 1 h prior to application of the drug suspension. Propylene glycol pre-treatment showed that percutaneous permeation of caffeine was significantly enhanced; therefore after subtracting the effect of propylene glycol on the penetration of caffeine, geraniol showed an enhancement ratio of 1.76 (which was 15.7 before subtraction) followed by (+)-neomenthol with a 1.52-fold increase (13.6-fold before subtracting) [57].

α-Terpineol was an effective enhancer for the percutaneous penetration of hydrocortisone (6.65-fold) and triamcinolone acetonide (2.47-fold). Propylene glycol did not significantly increase the amount of hydrocortisone or triamcinolone acetonide transported through the skin. These studies indicated that terpenes capable of hydrogen bonding are more effective penetration enhancers for hydrophilic drugs such as caffeine and hydrocortisone than for lipophilic drugs such as triamcinolone acetonide [57].

Gels containing different concentrations of tetrahydrogeraniol (the main chemical constituent of rose and ylang ylang oils) with or without different concentrations of propylene glycol were tested for their efficacy to enhance the permeation of 5-fluorouracil, a hydrophilic compound, across excised abdominal rat skin. Tetrahydrogeraniol, like geraniol, is also an acyclic monoterpene with the same structural formula, although its double bonds are saturated. Significant differences among 2, 5, 8 and 10% (w/w) concentrations of tetrahydrogeraniol formulations were found indicating that the permeation of 5-fluorouracil is dependent on the concentration of tetrahydrogeraniol present. Maximum flux was obtained at a concentration of 8% (w/w) tetrahydrogeraniol, while propylene glycol did not exert any major synergistic effect [55].

### 3.6. Sesquiterpenes

Sesquiterpenes are relatively large molecules [41] and are isolated from the higher boiling point fractions of commonly used essential oils. The penetration enhancing abilities in human skin of sesquiterpenes, chosen for their low toxicity and low coetaneous irritancy, were investigated using the hydrophilic drug, 5-fluorouracil as the model drug [45].

The selected sesquiterpenes included the following:

– Hydrocarbons: (+)-longifolene, β-caryophyllene, (+)-aromadendrene and (+)-β-cedrene;
– Alcohols: (−)-isolongifolol (synthetic derivative), (−)-guaiol, (+)-cedrol, (−)-α-bisabolol, farnesol and nerolidol;
– Others/miscellaneous: β-caryophyllene oxide and (+)-cedryl acetate which are both synthetic derivatives.

Epidermal membranes were treated with 150 to 200 μL of enhancer or enhancer formulation for 12 h. Some of the selected sesquiterpenes (*i.e.*, (−)-isolongifolol; (−)-guaiol; (+)-cedrol; β-caryophyllene oxide; (+)-cedryl acetate) were a solid at 32 °C and were therefore delivered in saturated dimethyl isosorbide as vehicle. A two-fold increase in 5-fluorouracil transport was seen with the hydrocarbon sesquiterpene compounds. Increased pseudo-steady-state 5-fluorouracil flux was observed with the cumulative permeation profiles, although in numerous occasions the lag-times were increased. The authors attributed the increased lag-times to the slow redistribution of the enhancers within the stratum corneum which gives rise to gradual increases in membrane permeability during the early stages of the diffusion process. Weak enhancement was observed with the sesquiterpene alcohol compounds in saturated dimethyl isosorbide solutions. 5-Fluorouracil absorption was best improved by the liquid sesquiterpene alcohols, with nerolidol being the best enhancer [45].

β-Caryophyllene oxide and (+)-cedryl acetate significantly improved the absorption of 5-fluorouracil, however, they were found to be less effective enhancers than farnesol and nerolidol (after allowing for the effect of dimethyl isosorbide), which were thought to be due to their slightly more compacted structure. Cornwell and Barry [45] suggested that the sesquiterpenes disrupt the intercellular lipid bilayers in the stratum corneum, thus improving 5-fluorouracil diffusivity, and that some of the compounds also increase 5-fluorouracil partitioning. Undesirably, the effects of the sesquiterpene enhancers were found to be poorly reversible due to the slow release of the enhancers from the stratum corneum [45].

Due to the absence of definite hydrocarbon tails in the cyclic compounds (−)-isolongifolol, (−)-guaiol and (+)-cedrol, they had poorer abilities to disrupt the lipids and thereby showed the weakest enhancing activities of the alcohols investigated. (−)-α-Bisabolol would align better within the lipid domain as a monocyclic alcohol and was of intermediate activity. With structures suitable for disrupting lipid packaging, nerolidol and farnesol (acyclic alcohols) were found to be the best enhancers. Comparing their structures showed that changing from a primary to a tertiary alcohol increased enhancer activity noticeably [45].

Nerolidol was found be the best penetration enhancer between four terpenes (*i.e.*, limonene, thymol, fenchone and nerolidol) for four model drugs including nicardipine hydrochloride (most hydrophilic), hydrocortisone, carbamazepine and tamoxifen (most lipophilic) [58]. The *in vitro* skin permeability studies were performed on female hairless mice skin in gel formulations compared to a control group which consisted of all the formulations’ components except the terpene enhancer. The efficacies of the terpenes were determined by comparing the following parameters: enhancement ratios (model drug flux with terpene in gel divided by the model drug flux without terpene in gel-control), cumulative amount of the model drug in the receptor after 24 h and skin content after 24 h. Nerolidol was followed by limonene and then thymol in terms of penetration enhancement of the model drugs. Fenchone showed the lowest increase in flux for all the model drugs. Limonene’s efficiency relative to thymol and fenchone was attributed to its higher thermodynamic activity in the gel as it was not completely soluble in the gel formulations at 2% concentrations. This indicates the influence that the composition of the gel formulation has on the enhancing activity of the terpene enhancers [58].

In addition, the skin content of the model drugs relative to the control group was found to be different for the different model drugs. Skin content of nicardipine hydrochloride was found to be the highest with limonene, followed by nerolidol, fenchone and thymol. No significant increase in skin content of hydrocortisone was found relative to the control with any of the terpenes. Skin content of carbamazepine was significantly increased by nerolidol, limonene and thymol; whereas fenchone significantly lowered carbamazepine’s skin content. With the model drug, tamoxifen, it was found that the terpenes did not have major effects on any of tamoxifen’s percutaneous permeation parameters relative to the control [58].

## 4. Fixed Oils/Fatty Acids

Fatty acids are composed of an aliphatic hydrocarbon chain, which can either be saturated or unsaturated, and a terminal carboxyl group. Fatty acids are known skin permeation enhancers and are regarded as non-toxic and safe for topical use [59].

### 4.1. Fish Oil

Fish oils are different from other oils mostly due to their unique array of fatty acid contents and the high degree of unsaturation of their fatty acids. Typically, over 90% of the refined oil consists of triglycerides and the remainder of monoglycerides, diglycerides, other lipids (e.g., phospholipids) and unsaponifiable matter (e.g., sterols, glyceryl ethers, hydrocarbons, fatty alcohols, vitamin A, D and E) [60]. The acid part of the glycerides is mainly made up of numerous unsaturated fatty acids which include eicosapentaenoic acid (EPA) and docosahexaenoic acid (DHA) [61].

Cod-liver oil is obtained from the fresh liver of cod [61]. During the refining of medicinal cod-liver oil, a fatty acid extract is obtained. About 17% of the extract consisted of saturated acids, primarily palmitic acid (10.4%), with the rest of the extract being unsaturated fatty acids such as oleic acid (15–16%), DHA (11.9%), gondoic acid (9.4%), EPA (9.3%), gadoleic acid (7.8%), palmitoleic acid (6.4%) and *cis*-vaccenic acid (4.4%). It was found that fatty acid extract of cod liver oil enhanced the permeability of hydrocortisone through hairless mouse skin and was concentration dependent, although the testing of unsaturated fatty acids showed much larger enhancement potency than the extract in the following order: palmitoleic acid > *cis*-vaccenic acid > EPA > DHA > oleic acid. Results indicated that the enhancement effect of the extract was linked to the unsaturated portion of the fatty acids. In a separate experiment it was found that pure cod-liver oil in propylene glycol did not increase the hydrocortisone permeation through the skin; thus indicating that the unsaturated fatty acids have to be in the free form to be able to act as skin penetration enhancer [61].

A subsequent study showed that when the fatty acid extract from cod-liver oil was added to propylene glycol saturated with acyclovir, a 50- to 70-fold increase in the drug’s flux was observed depending on the concentration (5%, 10% and 30% (w/w)) of the fatty acid extract. Interestingly, the lowest concentration showed the highest enhancement of the skin penetration of acyclovir [62].

### 4.2. Fatty Acids from Algae

“Algae” can be described as chlorophyll-bearing organisms including their colorless relatives, which are thalloid meaning they lack true roots, stems and leaves/leaf-like organs [63,64]. Green algae (Chlorophyta) fall in the group of eukaryotic algae with cells that contain membrane-bounded nuclei, which have chloroplasts surrounded only by the two membranes of the chloroplast envelope. *Botryococcus braunii* belongs to the Chlorophyceae class, which is one of the four important classes in the Chlorophyta family [64].

*Botryococcus braunii* is a freshwater species and can be classified as green algae [63], which is relatively widely distributed in ponds and lakes. The effect of fatty acids namely palmitic acid, oleic acid, linoleic acid and linolenic acid extracted from *B. braunii* on flurbiprofen’s absorption was studied [65]. *In vitro* Franz cell skin permeation studies and *in vivo* techniques with Wistar rats as the animal model were performed. The skin was pre-treated with 3% (v/v) *B. braunii* extract or each individual fatty acid in 25% propylene glycol/pH 7.4 buffer solution for 30 min during *in vitro* and *in vivo* studies and the flurbiprofen was subsequently applied in a hydrogel drug vehicle. The permeation of flurbiprofen was increased 2.6-fold compared to the control after pre-treatment with the *B. braunii* extract. It was suggested that the fatty acids in the *B. braunii* extract disrupts the structure of the skin and increase drug partitioning into the stratum corneum [65].

It was found that pure unsaturated fatty acids were significantly more efficient penetration enhancers than the *B. braunii* extract. Results obtained after pre-treatment with a simulated *B. braunii* extract were compared to the natural extract, and it was found that the flux of both were almost the same. This indicated that the free fatty acids in *B. braunii*, rather than the other compounds (*i.e.*, hydrocarbons, carotenoids and chlorophyll), are the most important components responsible for enhancing permeation of flurbiprofen into and across the skin [65].

Nevertheless the *B. braunii* extract showed a less irritant potential compared to the pure fatty acids. Interestingly, the simulated mixture disrupted the skin layer (based on measured transepidermal water loss and scanning electron microscopy images), and it was thought that other components present in the extract produced a buffer effect to reduce skin irritation caused by the fatty acids [65]. The authors concluded that *B. braunii* can serve as a safe and inexpensive skin penetration enhancer of drugs [65].

### 4.3. Phospholipids

Phospholipids are complex lipids containing backbone structures to which fatty acids are covalently bound. They are essential components of cell membranes and glycerophospholipids (phosphoglyceride/glycerol phosphatide) are members found in this group. The parent compound of glycerophospholipids, phosphatidic acid, is found in small amounts in the majority of natural systems. A range of polar groups are esterified to the phosphoric acid moiety of these molecules. When a hydroxyl-containing organic molecule becomes esterified to phosphatidic acid’s phosphate group, phosphatides are formed. Phosphatidylcholine (commonly known as lecithin) and phosphatidyl-ethanolamine are phosphatides containing choline and ethanolamine, respectively, and are two of the most common constituents of biological membranes. Glycerol, serine and inositol are some of the other common head groups found in phosphatides [66].

Phospholipids applied topically can generally be considered as safe [67,68] since they are degraded within the skin [69]. Results also indicated that they are milder on the skin than unsaturated fatty acids [70].

*In vitro* studies on excised rat skin showed that hydrogenated soya phospholipids (containing approximately 30% phosphatidylcholine and 70% phosphatidylethanolamine; iodine value approximately 6%) in aqueous gel form enhanced the penetration of sodium diclofenac as well as the amount of accumulated diclofenac in the skin tissue. This enhancement was due to accelerated penetration of diclofenac through the stratum corneum, rather than alteration in the distribution of the drug. *In vivo* results were consistent with the *in vitro* results, as a higher accumulation of diclofenac in the tissue gave rise to a higher plasma concentration of diclofenac [71].

*In vitro* studies on hairless mice skin indicated that the transdermal permeation of bunazosin hydrochloride, theophylline and isosorbide dinitrate was enhanced to different degrees when egg lecithin (1% (w/w)) was dissolved in the vehicle, propylene glycol. This enhancing effect was also observed with *in vivo* studies in male rabbits where higher plasma levels of bunazosin were obtained when lecithin was added to propylene glycol at a concentration of 3% (w/w) [72].

In another study it was found that the percutaneous penetration of flufenamic acid was enhanced when dispersed in phosphatidylcholine (from soybean) and even further enhanced in phosphatidylcholine/glycosylceramide (from soybean) disperse systems. It was, however, found during the *in vitro* study that the enhancing effect of the phosphatidylcholine-dispersion depended on the amount of phosphatidylcholine present in the system, as no significant difference among the lipid-free suspension and the phosphatidylcholine containing dispersions was seen when a concentration of 40 µmol/mL was exceeded. A maximal enhancement for flufenamic acid from the phosphatidyl-choline/glycosylceramide dispersion was observed when the ratio was 9:1. When phosphatidylcholine-dispersions with 30% propylene glycol and 30% glycerol in the same buffer solution were prepared, it was found that the penetration of flufenamic acid was significantly and insignificantly increased, respectively. In contrast, pre-treatment for 12 h with flufenamic acid free lipid dispersions showed no statistically significant change in the penetration of the drug. It was concluded that the lipid disperse systems enhanced the penetration of flufenamic acid by two mechanisms: (1) lipids could alter the permeability of the stratum corneum by having a direct effect and/or (2) solubility of the drug could be increased [73].

The effect of phospholipids on percutaneous absorption of naproxen was investigated. During this study hydroalcoholic, aqueous and propylene glycol gels were prepared with some formulations containing levo-α-phosphatidylcholine (EPC, 60% from fresh frozen egg yolk) and levo-α-phosphatidylcholine (SPC, 60%, from soybean) phospholipids. Franz-type diffusion cells were used during the permeation studies with female cadaver abdominal skin. It was concluded that the percutaneous absorption of naproxen was increased when co-solvents such as propylene glycol or ethanol was included in the formulations containing EPC and SPC; whereas EPC and SPC in aqueous gels were not able to increase the penetration of naproxen [68].

Furthermore, it was found that EPC in the 32% ethanol solution increased naproxen’s permeation compared to the control; whereas in the 8% ethanol solution the permeation of naproxen was decreased compared to the control [68]. This indicated that the enhancing effect of phospholipids needs the presence of a certain amount of ethanol. It was thought that ethanol could cause this effect by two mechanisms: (1) ethanol causes the phospholipids to penetrate into the skin by enhancing the fluidity of the skin lipid multilayer, and/or (2) ethanol loosens phospholipid vesicles which cause phospholipids to penetrate into the skin and disrupt the stratum corneum’s bilayer [68,74].

*In vitro* studies indicated in general that the more soluble a phospholipid is in propylene glycol, the higher the percutaneous penetration is of indomethacin. It was found that phospholipids enhanced the penetration of indomethacin (from propylene glycol solution) in the following order: phosphatidylglycerol (egg yolk) > phosphatidylethanolamine (egg yolk) > phosphatidylcholine (egg yolk) > phosphatidylserine (soybean) > phosphatidic acid (egg yolk) > phosphatidylinositol (soy bean) > control > sphingomyelin (egg yolk) [69]. By using Attenuated Total Reflectance-Fourier Transform Infrared Spectroscopy (ATR-FTIR), it was found that phospholipids with an unsaturated acyl chain increases lipid fluidity in the stratum corneum, thereby enhancing percutaneous penetration of drugs such as prednisolone [67].

Percutaneous penetration of drugs, such as lipophilic prednisolone, is enhanced by phospholipids containing an unsaturated acyl chain (e.g., phosphatidylglycerol, phosphatidylcholine and phosphatidylethanolamine from egg yolk; phosphatidylcholine and phosphatidylglycerol from soybean) in their hydrophobic groups; whereas phospholipids containing only saturated acyl chains (e.g., hydrogenated phosphatidylcholine) in their hydrophobic groups are efficient for inhibiting percutaneous penetration [67]. The same was seen when numerous derivatives of phosphatidyl-glycerol, phosphatidylcholine and phosphatidylethanolamine was investigated for their penetration enhancing effects on indomethacin [70].

Yokomizo and Sagitani [70] also determined that phospholipids with a lower transition temperature (T_m_) increased the percutaneous penetration of indomethacin. It was thought that this lower value in transition temperature could cause the phospholipids to be incorporated more easily into the viable cells via intercellular lipids in the stratum corneum. This will disrupt the lamellar structure and raise the fluidity of the cell membranes’ lipid bilayer, making a lipophilic route for indomethacin to penetrate through.

### 4.4. Vesicular Carriers

Novel vesicles prepared by phospholipids and natural penetration enhancers such as terpenes have been described for transdermal drug delivery. Liposomes are microscopic vesicles that consist of aqueous compartments surrounded by membrane-like lipid layers. These lipid layers consist of amphiphilic phsopholipids with a hydrophilic head and a lipophilic tail [1]. Different penetration enhancers (including cineole) were evaluated for their ability to produce elastic vesicles with soy lecithin and their subsequent effect on the *in vitro* transdermal delivery of minoxidil [75]. No permeation of minoxidil through the whole skin was observed for both the classic liposomes (soy lecithin and dicetylphosphate) and the liposomes containing the penetration enhancers. However, the skin deposition of minoxidil was improved by the vesicles containing penetration enhancers when compared to classic liposomes and ethanolic solutions of the penetration enhancers, thus improving percutaneous drug delivery [75].

Invasomes investigated as penetration enhancers were composed of soybean phosphatidylcholine, ethanol and a mixture of terpenes (*i.e.*, cineole, citral and *d-*limonene). It was found that invasomes containing 1% of the terpene mixture effectively delivered the highly hydrophobic drug, temoporfin, into the stratum corneum and the deeper layers of the skin when compared to liposomes containing 3.3% ethanol or liposomes without ethanol [76].

Novel vesicular carrier systems consisting of phospholipids, water and high concentrations of ethanol are known as ethosomes. Ethosomes were found to enhance the delivery of drugs such as minoxidil and testosterone in terms of both the flux as well as drug concentration in the skin when compared to the control systems [77].

## 5. Polysaccharides

Carbohydrates are generally the most abundant class of organic molecules found in nature and can be classified into three groups, one of which is polysaccharides. Polysaccharides are polymers of simple sugars and their derivatives can be branched or linear and may possibly consist of hundreds or thousands of monosaccharide units [66].

### 5.1. Chitosan and Derivatives

Chitosan is a cationic polysaccharide obtained by the deacetylation of chitin, which occurs naturally in the exoskeletons of marine organisms such as crab and shrimp [78,79]. Chitosan has poor solubility at physiological pH values (pH above 6.5), while its derivatives such as *N*-trimethyl chitosan and mono-*N*-carboxylmethyl chitosan, have improved solubility over a wide pH range. A study was performed wherein two *N*-trimethyl chitosans (TMC) with different degrees of quaternization (DQ of 38.8% and 67.2%) was synthesized and will be indicated as TMC40 and TMC60 respectively. *In vitro* as well as *in vivo* permeation studies on full-thickness mice skin and in rabbits, respectively, were conducted by applying testosterone gel formulations. During both *in vitro* and *in vivo* studies, gels containing 5% TMC40 and TMC60 both increased the transdermal absorption of testosterone noticeably when compared to the control without enhancer, although TMC60 proved to be more significant than TMC40 at the same concentration. TMC40 showed, however, no significant difference compared to testosterone gels containing 2% Azone^®^. Therefore, the results proposed that the enhancing effect of TMCs increases along with an increase of DQ [80].

Chitosan appears to interact with negative charges in the skin to improve drug (in this case doxorubicin) diffusion into the deeper layers of the skin [81]. Other studies were also performed to investigate the mechanisms by which chitosan and its derivatives enhance transdermal penetration [76]. With ATR-FTIR spectroscopy the transformation of the secondary structure of keratin in the stratum corneum (mice skin) after treatment with chitosan, *N*-trimethyl chitosan and mono-*N*-carboxylmethyl chitosan was investigated. This loosens the accumulative structure of keratin leading to a larger degree of freedom for carbon movement to improve transdermal drug permeation [79,80].

It was found that different molecular weights of *N*-arginine chitosan derivatives with different degrees of substitution have the capability to enhance the transdermal delivery rate of adefovir across abdominal mice skin. *N*-arginine chitosan derivatives were also found to be 1.83, 2.22, 2.45 times more effective as percutaneous transport enhancer than Azone^®^, eucalyptus and peppermint oil, respectively [82].

### 5.2. Aloe vera Gel/Juice

*Aloe vera* is a member of the Asphodelaceae family with a long history as traditional folk remedy and is most commonly used to treat conditions such as constipation, arthritis, blood pressure problems, burns, diabetes, eczema, psoriasis and skin cancer [83,84,85]. Polysaccharides and lectins present in the inner pulp or gel of the leaves are considered to be the most important components [84].

The *in vitro* skin permeation enhancement potential of *A. vera* leaf gel extract, using porcine ear skin membranes, has been studied by Cole & Heard [86]. A series of drugs with different lipophilic values and molecular weights were used which included caffeine, colchicine, mefenamic acid, oxybutynin and quinine. Saturated solutions of these compounds were prepared in deionized water and *A. vera* juice (“standard strength”) at 32 °C in order to test the compounds’ solubility in both and to determine their transport across skin in Franz diffusion cells [86]. The *in vitro* studies showed that *A. vera* has drug permeation enhancement properties across the skin. Physiochemical properties such as the calculated octanol-water partition coefficient/drug lipophilicity and molecular weight of the model drug compounds were investigated and they were found to influence the enhancement properties of the *A. vera* material. In addition, it was found that a significant proportion of *A. vera* constituents permeated the skin together with the model drug compound [86].

Interestingly, *A. vera* gel had a higher permeation enhancement effect on drugs with a higher molecular weight. This was explained by the fact that a drug with a larger molecular weight effectively blocks the permeation routes allowing increased possibility for the drug to interact with the enhancing factor and complex with it prior to being transported across the skin. It was further found that “double strength” *A. vera* at a concentration of 3% (w/v) enhanced the permeation of quinine significantly higher when compared to the “standard” strength [86].

Contrasting results were found with ketoprofen as the model drug, when skin was pre-treated with *Aloe vera* juice. Pig ear skin incorporated into Franz-type diffusion cells was pre-treated (1 h) with either of the following: Commercial *A. vera* juice, commercial *A. vera* juice followed by messaging, boiled and cooled *A. vera* juice, deionized water as negative control and tea tree oil as the positive control. The cells were then dosed with 500 μL of a saturated solution of ketoprofen in PEG-400 (polyethylene glycol). It was found that the difference between the pre-treatment with either of *A. vera* juice, massaging of *A. vera* juice or boiled *A. vera* juice was statistically insignificant compared to water [87]. The article published by Cole and Heard [86] suggested that solute drugs investigated in their study complexed with the enhancing factor before being transported across the skin; however in the work done by Ballam and Heard [87] ketoprofen could not interact with the *A. vera* phytochemicals in the same manner (not used ‘within-vehicle’). It was concluded that due to the constituents of *A. vera* being dependable on a wide range of factors, such as climate, location and soil, *A. vera* from other sources may give rise to different results [87].

## 6. Miscellaneous

### 6.1. Capsaicin

**Figure 9:**
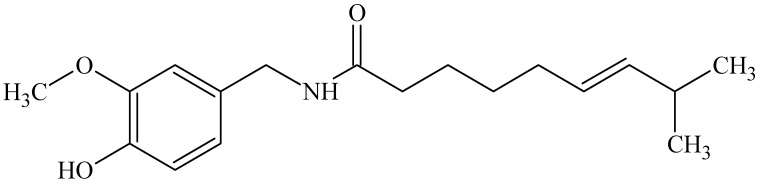
Chemical structure of capsaicin.

Capsaicin (*trans-*8-methyl-*N*-vinillyl-6-nonenamide) (Figure 9) is an alkaloid derived from hot chili peppers, belonging to the genus Capsicum of the Solanaceae family [88,89]. The chemical structure of capsaicin has some similarities to the structure of Azone^®^. Both contain a ring at one end of a long alkyl chain, although their partition behavior is different with a log P value of 3.31 and 7.82 for capsaicin and Azone^®^, respectively. Capsaicin is therefore probably much better absorbed percutaneously than Azone^®^ [90]. The penetration enhancing effects of capsaicin on naproxen was investigated with *in vitro* experiments employing full-thickness, female human skin and an *ex vivo* perfused rabbit ear model. Skin samples were treated with 50 μL/cm^2^ of capsaicin in ethanol for 2 h prior to the diffusion studies, then left unoccluded so the ethanol could evaporate. It was found that capsaicin enhanced the permeation of naproxen through full-thickness skin approximately 2-fold. When comparing the fluxes of naproxen obtained with the ex vivo perfused rabbit ear model, capsaicin had an enhancement ratio of 3.8 when compared to the control. It was also determined that the fluxes of naproxen were lower than those found in human tissue, although the effect is of a comparable magnitude [90].

After microscopical evaluation of the skin, no overall changes were observed in the structural features of the stratum corneum with little evidence of skin thickening. The authors therefore thought the enhancement effect of capsaicin could not be attributed to structural damage to the outer layers of the skin. It is possible that capsaicin reduces the diffusional resistance of the intercellular domains by inserting itself into the lipid bilayers within the intercellular channels thereby disrupting their stacking. Capsaicin also lowers substance P [89], hence it was suggested that topical analgesic formulations can be created in combination with naproxen where the capsaicin will also act as an enhancer for naproxen [90].

### 6.2. Vitamin E

Research showed that vitamin E (α-tocopherol) enhanced the permeability coefficient of radiolabeled hydrocortisone with an average enhancement factor of 1.81 through excised human cadaver skin. In addition it was found that there was a reduction of lag time in skin samples treated with vitamin E. This moderate improvement in the permeability of the stratum corneum can be attributed to the restricted insertion of vitamin E in the ceramide-rich bilayer structure. Therefore, the permeability is affected due to the alteration of the membrane characteristics which is assumingly due to the disordering of the gel phase lipids [91].

## 7. Conclusions

The fact that the transdermal drug administration route offers so many advantages over oral administration of drugs has stimulated research to find ways to overcome the barrier function of the skin. One of the approaches to enhance drug permeation across the skin includes the incorporation of skin penetration enhancers into drug formulations. Unfortunately some skin penetration enhancers are toxic and a need therefore exists for discovery of safe and effective skin penetration enhancers which led to screening of natural compounds for this purpose. The aim of this review article was to summarise research done on skin penetration enhancers of natural origin. Many chemical compounds extracted from natural sources showed potential as skin penetration enhancing agents. It was further observed that the effectiveness of the penetration enhancers depends not only on their concentration in the formulation, but also on the physico-chemical characteristics of the drug to be transported through/into the skin layers. It can be concluded from the literature that natural penetration enhancers will play a major role in developing effective transdermal products in future.
